# Demystifying Cancer Immunotherapy for Lay Audiences

**DOI:** 10.3389/fimmu.2019.02488

**Published:** 2019-10-18

**Authors:** Kiara Ellis, Christopher A. Pennell

**Affiliations:** ^1^Office of Community Engagement and Education, Masonic Cancer Center, University of Minnesota, Minneapolis, MN, United States; ^2^Center for Immunology, Department of Laboratory Medicine and Pathology, University of Minnesota, Minneapolis, MN, United States

**Keywords:** cancer, community, engagement, immunotherapy, outreach

## Abstract

Immunotherapy is now mainstream. Advertisements are ubiquitous in print and visual media for immune based-therapies for various conditions and diseases. Smaller companies that develop novel immunotherapies are often quickly acquired by larger companies. More and more clinical trials are open for immune-based therapies, particularly for immune checkpoint blockades. As such, immunologists need to engage the public in conversations about the strengths and limitations of immunotherapy, and the necessity of research in propelling the field further. In this article, we discuss approaches we have taken to convey key concepts in immunology and cancer immunotherapy to non-scientists and health care professionals without expertise in immunology. Although the devil is always in the details, basic concepts in immunology and immunotherapy can be readily conveyed using stories and analogies, some of which we present here.

## Introduction

The need to inform the public about immunotherapy is more important than ever, as immunotherapy is now a key driver of cancer care and precision health. Here we describe community outreach approaches in immunology and cancer immunotherapy we developed for the Masonic Cancer Center (MCC), an NCI-designated comprehensive cancer center at the University of Minnesota.

## Know Your Target Audience

Time is precious. Don't waste it by giving the audience a “one size fits all” rote presentation or one you would give your peers. Identify topics that are likely of greatest interest to your audience by asking representatives beforehand what their most important issues or questions are. Meld your expertise with the needs and background of your audience and tailor the presentation specifically to them. The audience should leave with actionable knowledge and the belief that their time was well spent.

Our discussions of immunology basics don't differ much between disparate ethnic groups but our discussions of how to apply immunology do. These are tailored to the needs of the group. For example, cervical cancer rates are higher for American Indian, African American, Hmong, and Hispanic women in Minnesota than for others (1–3). In meeting with these groups, we often focus on how vaccines work, how vaccination against human papillomavirus (HPV) reduces the risk of cervical cancer, and the need to increase cervical cancer screening for early detection and a subsequent reduction in cancer mortality (1).

Be aware of the audience's range of knowledge in science and medicine. If you are updating health care workers on checkpoint blockade therapy or chimeric antigen receptor-transduced T-cells, you can assume a baseline knowledge of the immune system and focus on the specific strengths and limitations of these therapies. If you are speaking to a broad audience, assume an eighth-grade average reading level and a cursory knowledge of immunology and cancer (4). We developed a series of animated videos that includes Cancer 101; this describes cells, how cancers can form, and how to minimize risks (5). Because this video is appropriate for both adult and youth audiences, we find it useful to show at the outset of presentations.

Maximize visuals and graphics on slides while minimizing text. Use analogies, simple language, and avoid jargon whenever possible. When it is not possible to avoid a technical term, define, and explain it clearly before weaving it into your story. Know the physical layout of the venue in which you will speak. This includes its audio and visual equipment, lighting, and acoustics.

## Engage the Audience

Consider using experiential activities accessible to broad audiences. This will provide participants who learn by visual, auditory, and kinesthetic methods opportunities to access and retain the information. We invested in wireless polling devices that allow the audience to respond to questions posed by the presenter in real time. This permits the presenter to gauge the audience's readiness to move on to the next section. Because these data measure impact and collect information anonymously in a non-threatening way, we derive information from communities less inclined to respond to surveys.

What follows is the story we typically tell adult, lay audiences about cancer immunotherapy. This is not meant to be an inclusive review; rather it is one example of how to explain immunology and cancer immunotherapy. We focus on recent advances in T-cell based immunotherapy because these are more topical than well-established monoclonal antibody-based therapies such as Herceptin for breast cancer and Rituximab for B-cell lymphomas (6, 7).

## Immune Activation

We begin by describing how molecules, cells, tissues, and organs in the body work coordinately in systems to achieve particular functions. Most everyone is aware of the digestive system, so we begin there by saying the digestive system processes food and absorbs nutrients and water. People are generally less aware of how the immune system functions, so we start by saying the immune system maintains homeostasis throughout the body. When that balance is perturbed by injury, infection, or disease, the immune system is activated. Under normal physiological conditions in a healthy individual, an activated immune system restores homeostasis by eliminating the infection, healing the injuring, or eradicating the disease; the immune system then itself returns to homeostasis. What flows naturally from this introduction are discussions of what turns on and off immune responses.

We show pictures of red and white blood cells and note that white blood cells are part of the immune system. White blood cells become activated and start an immune response when their receptors signal that an infection/injury/disease has occurred. At this point we define a receptor. We show an animated slide that likens receptors and the signals they deliver to the electromagnetic waves received by home satellite dishes and the resultant images they relay to monitors. The external signal received by the receptor/satellite dish is conveyed to the cell/living room via an internal signal cascade/cable network. Physiologically, receipt of this internal signal leads to a change in the white blood cell's activity and the beginning of an immune response. Questions that logically follow this description include: what are these immune-activating signals, where do they come from, how are they recognized, and how do they mediate changes in cell function?

We next note that signals indicative of an infection typically come from the pathogen itself and so are externally-derived. Before going further, we define pathogen as a disease-causing entity. Collectively pathogen-associated molecules that induce immune responses are called stranger signals and include molecules we cannot make ourselves. In contrast, danger signals are internally-derived molecules our bodies make in response to tissue injury or disease. Danger signals are not normally accessible to the immune system but are released when a cell is damaged or ruptured or stressed. Stranger and danger signals typically indicate something deleterious has occurred that requires an activated immune system to resolve. The receptors on immune cells that recognize stranger and danger signals have coevolved with the cells' abilities to contain or eliminate physiological insults (8).

The immune system has a spectrum of molecular and cellular mechanisms that maintain homeostasis. Innate immune responses reside at one end of the spectrum and acquired immune responses at the other. Innate immune responses are elicited by stranger and danger signals, cause inflammation, and recruit leukocytes that can non-specifically eradicate pathogens. That is, innate responses can eliminate groups of pathogens but do not distinguish between individual pathogens within the group. Innate immune responses also trigger acquired immunity; these responses take longer to resolve infections because pathogen-specific immune cells are initially present at low frequencies (≤10^−5^) and take time to expand to sufficient numbers to control the disease (9). Acquired responses are specific to molecules unique to the disease-causing organism. The advantages of acquired immune responses include this specificity and long-lived memory responses to prevent recurrent infections of the same pathogen. The net result is that activated white blood cells can destroy invading bacteria, kill virally infected cells before viruses are released, and eliminate nascent tumors.

## Immune Surveillance

Immune surveillance refers to the immune-mediated elimination of nascent malignancies before they become clinically apparent. This occurs constantly and perhaps is the last barrier a cell must breach before becoming malignant (10, 11). By definition, cancers have escaped immune surveillance. And this stymied the field of cancer immunotherapy for over a century. Immunologists long recognized the immune system could be exploited to treat cancer because of four key characteristics: specificity, potency, memory, and adaptability. Specificity is the holy grail of cancer therapy because it widens the therapeutic window by reducing off-target toxicity. Potency permits relatively small numbers of cells to mediate curative responses. Memory minimizes the potential for recurrence. And adaptability counters the genetic instability of many tumors; tumor cells that express altered proteins (neoantigens) arising from ongoing mutations can be recognized as foreign and eliminated immunologically. Before we can discuss how malignant tumors evade immune surveillance, though, we must consider how tumors arise in the first place.

## The Odds Are Not in Our Favor

Let's do the math. For one cell to become two, it must copy all of its contents. These include proteins, lipids, carbohydrates, and nucleic acids. Nucleic acids are DNA and RNA and are the genetic storage, retrieval, and information transfer systems of the cell. All of the information encoded by a cell's DNA is called its genome. The genome is akin to a cookbook filled with recipes cells follow to function properly. There are only four different letters in a cell's cookbook, but each cookbook contains 12.8 billion total letters (6.4 billion base pairs per human diploid genome × 2 nucleotides/base pair). It is estimated that the average human adult has about 37 trillion cells (12). If we assume a daily turnover rate of about 0.5% (200 billion cells), then about 2.5 trillion billion (10^21^) nucleotides must be copied every day. To put this differently, the DNA in a single human cell is about 2.2 m long (340 pm/base pair × 6.4 billion base pairs/human diploid genome). The length of DNA copied every day is therefore approximately 440 billion meters, which is almost three times the distance from the earth to the sun. Copying errors are inevitable with such large numbers, and these copying errors can alter the recipes in the cookbooks and lead to cancer.

## What is Cancer and How Do We Get It

Cancer is defined as a population of cells that grow uncontrollably and invade local or distant tissues. Cancer arises from changes in DNA itself (genetic) or changes in how and when different regions of DNA are accessed (epigenetic). To carry the cookbook analogy further, the addition of the letter “b” to “tsp” increases the amount of an ingredient added from a teaspoon (tsp) to a tablespoon (tbsp). Single letter changes (i.e., point mutations) could have no, slight, or profound consequences, depending on the ingredient and recipe. Changes on a larger scale would be like tearing off the bottom half of one recipe and replacing it with the bottom half of a recipe from a different chapter (i.e., a translocation). Alternatively, the cell might inappropriately use one recipe (e.g., for crème brûlée) when it should have used another (e.g., sautéed liver and onions). This is called an epigenetic error (“epi” means above). The genetic material itself has not changed but the way it is used has. Epigenetic changes could have deleterious consequences for the host (e.g., if guests were promised crème brûlée for dessert but instead were given sautéed liver and onions).

Cellular mechanisms have evolved to minimize genetic mistakes, to correct mistakes once they are made, to provide redundancies to counterbalance loss-of-function mutations, to induce cell death if a cell acquires too many genetic lesions to copy its DNA successfully, and to eliminate nascent malignant cells via immune surveillance. Cancers ultimately evade all of these barriers typically by accumulating mutations and genetic lesions sequentially over decades (11, 13).

With this information we can now distill how someone gets cancer down to three ways. (*1) Old age*. Cancer is like a biological clock. The longer an individual is alive, the more s/he can acquire deleterious mutations from DNA copying errors or exposure to carcinogens (DNA-damaging agents) that can lead to cancer. (*2) Bad luck*. An individual can inherit mutated genes that predispose them to cancer, or they can be unintentionally exposed to sufficient doses of carcinogens to cause cancer. (*3) Lifestyle choices*. Cancer prevention and regular screening are likely the best ways to reduce one's risk for cancer. Prevention includes minimizing exposure to known carcinogens, being vaccinated against pathogens known to cause cancer, and eating foods rich in anti-oxidants and other known chemopreventive agents. Regular screening (e.g., colonoscopies) can detect cancer at earlier, more treatable stages. It is worth stressing that among old age, bad luck, and lifestyle choice, the only one we can control is our choice of lifestyles.

## Cancer Immunotherapy

**Figure 1:**
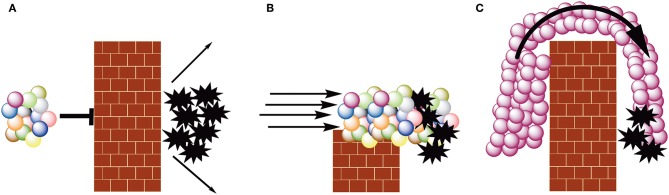
The spheres represent cells of the acquired immune system, with each color representing a different specificity. The brick walls represent tumor-induced immune suppression. The black, 10 point stars represent tumor cells. **(A)** Escape from immune surveillance. Malignant cells suppress immune effector cells. **(B)** Checkpoint blockade therapy. Inhibiting cancer-induced immune suppression via checkpoint inhibition permits tumor infiltrating lymphocytes to kill malignant cells. **(C)** CAR T-cell therapy. Autologous white blood cells (typically T- or NK-cells) are transduced with a CAR-encoding construct, rendering all the transduced cells specific for the same antigen. These cells are expanded to large numbers *ex vivo* and then infused into the patient.

Cancers co-opt normal biological processes to escape immune surveillance. We present these processes collectively as a wall between the malignant tumor and anti-tumor immune cells (Figure 1A). It has only been in the past 10–20 years that immunologists have begun to understand how cancers erect these walls: what the bricks and mortar are. Although this knowledge has led to many immune-based approaches for cancer therapy, most rely on one of two strategies.

The first is figuratively to reduce the height of the wall or compromise its integrity (Figure 1B). This permits tumor-infiltrating lymphocytes and other *in situ* immune effector cells to avoid suppression and eliminate malignant cells. Immunological approaches that fall into this class include checkpoint inhibitors. Checkpoints such as CTLA-4 and PD-1 suppress activated immune cells and allow them to return to homeostasis (14, 15). Some cancers engage these checkpoints and escape immune surveillance; monoclonal antibody-mediated inhibition of checkpoint signaling permits immune-mediated tumor cell death (16, 17). FDA-approved checkpoint inhibitors such as Yervoy (ipilimumab; anti-CTLA-4) and Keytruda (pembrolizumab; anti-PD-1) can profoundly increase survival for some patients with cancers such as melanoma and metastatic non-small cell lung cancer (18).

The second strategy is to induce such a strong immune response that it figuratively crashes over the wall, much like a tsunami breaching a seawall (Figure 1C). This approach relies on mass action: the number of immune effectors exceeds the number of immune inhibitors. This immune tsunami is typically created in three ways. The first is to use a therapeutic vaccine to elicit an anti-tumor response in the patient (19). This approach has had limited success primarily because the patient's immune system is systemically suppressed by disease and prior therapies. Figuratively this creates a hole in front of the wall making the barrier that much higher.

Discussions of cancer vaccines with lay audiences must address persistent misconceptions about the safety of vaccines. We suggest a multi-pronged approach. State that vaccines are among the biggest success stories in modern medicine. Show pictures of individuals infected with smallpox and pediatric polio victims in iron lungs; these images are likely to have the greatest impact. Show data regarding the dramatic declines in mortality due to vaccination and the eradication of smallpox in 1980 (20). Briefly describe how vaccines elicit pathogen-specific immune responses in the absence of disease; these responses then prevent disease by quickly eliminating the pathogen should it infect again. Note the 1998 publication that fueled the anti-vax movement has been discredited and retracted (21). This paper claimed that the measles/mumps/rubella vaccine was linked to autism in children. However, the data were irreproducible, and the lead author did not reveal that some of his research was funded by lawyers suing vaccine manufacturers. Acknowledge that while vaccinations often cause common local reactions (e.g., pain, swelling, and redness at the injection site), these are minor and transient and simply indicate recruitment of immune cells that subsequently will protect against infection from the pathogen targeted by the vaccine. Conclude that vaccines are a boon to humanity and that herd immunity protects children and immune-compromised individuals.

The second approach to create an efficacious anti-tumor response is to remove tumor-specific cells from the patient, grow them to large numbers in the laboratory, outside of the immune-suppressive environment of the patient's body, and then return them to the patient. This has had more success than the vaccine approach, but it is hampered by the difficulty in identifying truly tumor-specific immune cells in the patient (22). The third approach takes some of the patient's healthy white blood cells and genetically reprograms them to recognize and kill tumor cells, regardless of what the immune cells were born to recognize. The engineered autologous cells are expanded *ex vivo* and then infused in the patient. This approach has been a game changer for certain B-cell leukemias and lymphomas as patients with otherwise incurable diseases are alive today (23). These genetically modified cells are called CAR cells, where the acronym CAR stands for chimeric antigen receptor.

While cancer immunotherapy has enormous potential, we need to caution that providing false hope can be an unintended consequence of presentations like those we just described. The presenter has a moral and ethical obligation to note that many patients still do not respond favorably to cancer immunotherapy, and that it has other drawbacks. These include acute and chronic immune-related adverse effects, cost, and access. More research is needed to overcome these limitations.

## Conclusions

In many cultures, storytelling is the traditional method of teaching. In the Hmong culture, skills, customs, historical knowledge, and traditions are passed orally from generation to generation via rote learning, memorizing, and storytelling (24). Because humans are attuned to story-telling, we tell stories based on immunology and cancer immunotherapy that weave in facts with easily recognizable analogies. We typically begin talks on cancer immunotherapy with a picture taken in 2010 of five-year old Emily Whitehead, the first pediatric patient treated with CD19-specific CAR T-cells (23). The Whitehead family has allowed Emily's story to be told publicly to promote immunotherapy. We say that in 1960, Emily would have had a 10% chance of survival given her diagnosis of pre-B-cell acute lymphoblastic leukemia. But thanks to 50 years of research, her prognosis in 2010 was much better as her chances of long-term survival were 85–90%. Unfortunately, she relapsed following standard therapy and was near death with resistant disease in 2012. We then state we will return to Emily at the end of the talk, which we do after presenting the above material starting with immune activation and ending with cancer immunotherapy. At the end of our presentation we close the story loop by showing a picture of a healthy teenage Emily taken in 2019. At this point we present the limitations of immunotherapy, particularly CAR T-cell immunotherapy, and note that only more research will lead to improved outcomes with reduced off-tumor effects. We then have an open question and answer period followed by informal interactions with the attendees.

We routinely provide our slide decks to the attendees electronically and give them printed materials with contact information for MCC specifically and cancer immunotherapy in general. We have pamphlets printed in English, Hmong, Somali, and Spanish to reflect the demographics of our community. These outreach efforts are almost always well-received and leave attendees with the belief that their time was well spent.

## Author Contributions

KE and CP developed various community outreach activities related to cancer prevention and immunotherapy, and together outlined and wrote this article.

### Conflict of Interest

The authors declare that the research was conducted in the absence of any commercial or financial relationships that could be construed as a potential conflict of interest.
